# Determination of a Central Avascular Triangle within the Obturator Foramen: A Radioanatomic Study

**DOI:** 10.1371/journal.pone.0143642

**Published:** 2015-12-01

**Authors:** Krystel Nyangoh Timoh, Georges Bader, Arnaud Fauconnier, Vincent Barrau, Vincent Delmas, Cyril Touboul

**Affiliations:** 1 Department of Obstetrics and Gynecology, Hôpital Intercommunal de Créteil, University Paris Est, UPEC-Paris XII, Paris, France; 2 Department of Obstetrics and Gynecology and Reproductive Medicine, Hôpital de Poissy, University Saint-Quentin-en-Yvelines, Poissy, France; 3 Department of radiology, Centre cardiologique du Nord, Saint Denis, France; 4 URDIA EA 4465, Anatomy, Université Paris Descartes, Paris, France; 5 UMR INSERM U965: Angiogenèse et Recherche translationnelle, Hôpital Lariboisière, Paris, France; University of Brescia, ITALY

## Abstract

**Purpose:**

To map the vascular anatomy of the obturator foramen using fixed anatomic landmarks.

**Method:**

Twenty obturator regions were dissected in 10 fresh female cadavers after vascular blue dye injection in five cadavers (50%). Furthermore, 104 obturator regions were reconstructed by angiotomodensitometry from 52 women under investigation for suspected arterial disease. The anatomy of the obturator region was mapped by measuring the distance of vascular structures from the middle of the two branches of the ischiopubic bone, which were used as fixed landmarks.

**Results:**

The bifurcation of the obturator artery was at a mean (SD) distance of 30.0 mm (4.5) from the middle of the ischiopubic branch (MISP). The anterior branch of the obturator vessels was 15.2 mm (10.1) from the MISP. The posterior branch of the obturator vessels was 5.5 mm (4.0) and 23.6 mm (8.7) from the middle of the outer edge of the obturator foramen (MOE) and the MISP, respectively. Using 5° and 95° percentiles of these measurements we defined a central avascular triangle.

**Conclusions:**

Our data show that, beyond inter-individual variations, a central triangular avascular area can be identified in the obturator foramen between the posterior and anterior obturator artery using fixed landmarks.

## Introduction

Pelvic organ prolapse may occur in up to 50% of parous women and prolapse repair surgery is thus a major public health issue [1–3]. Among the various surgical approaches, vaginal procedures are particularly relevant in cases of anterior vaginal wall prolapse and indicated for recurrent prolapse or risk factors of recurrence [4]. The procedure involves the placement of an anterior transvaginal mesh which is commonly performed by a transobturator approach [5,6].

While the use of a transvaginal mesh has been shown to be both subjectively and objectively more effective than anterior colporrhaphy [4], many complications related both to the mesh and to the transobturator approach have been described [1,7,8]. The transobturator procedure involves inserting two needles in both obturator areas without visual control. Several cases of hemorrhagic complications following this procedure have been published [9–15]. In a previous anatomic study, we demonstrated that the posterior needle passes close to the obturator vessels [16]. However, the surgeon is unaware of the position of the needle as he/she is guided by palpation only. Although anatomic descriptions of the obturator area are available in textbooks [17], there is a lack of practical fixed surgical landmarks which could be useful for urogynecologic surgeons.

The aim of this study was therefore to map vascular structures of the obturator foramen using fixed anatomic landmarks to better understand the vascular environment within the obturator foramen with a view to reducing the risk of vascular complications during prolapse pelvic surgery.

## Material and Methods

Data were obtained from both anatomic study in ten cadavers and radiologic study in 52 women undergoing investigation for arterial disease. The study was approved by the French Research Ethics Committee (number: 2015-01-04). Written patient consent was not required for this strictly observational study. The scientific committee of the Department of Anatomy, University Paris Descartes, ensured that the anatomic subjects were obtained by body donation made during their lifetime with a written informed consent on file from those who donated their bodies.

### Radiologic study

**Fig 1:**
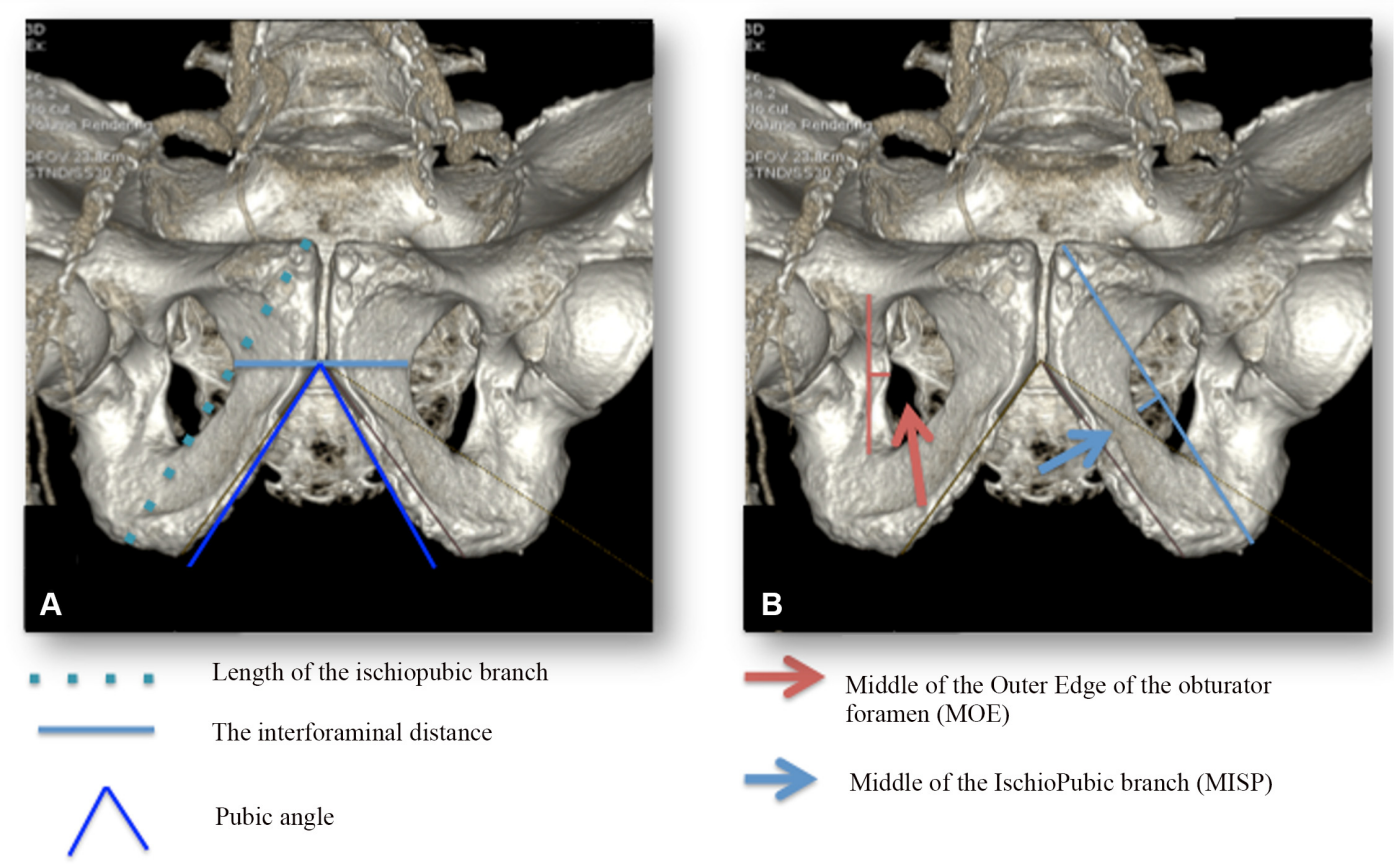
A & B: Bony measurements and bony landmarks on 3D angiotomodensitometry reconstruction. Pelvic bone is represented. We also show the main measurements realized: length of the ischiopubic branch, the interforaminal distance and the pubic angle. Bony landmarks chosen are the Middle of the IschioPubic branch (MISP) and the Middle of the Outer Edge of the obturator foramen (MOE).

We included 104 obturator regions from 52 women who were investigated for suspicion of distal arteriopathy at the radiology center “Centre Cardiologique du Nord (CCN)”. Standard axial, sagittal and coronal sections were performed using the 750 High Resolution CT scan (General Electrics Healthcare®). The post-treatment data were analyzed with the Advantage workstation. The arteries of the obturator foramen were reconstructed by angiotomodensitometry and checked for concordance with standard sections. The 3D reconstructions were performed by adaptive statistical iterative reconstruction (ASIR), 40% mode without filter. Spatial resolution was of 230 to 350 μm. We recorded anatomic descriptions and observations. Fig 1 shows the different landmarks and measurements obtained on 3D reconstruction. We first measured the bone limits of the obturator foramen, including the interforaminal distance (*i*.*e*. the smallest distance between the left and right obturator foramen), the pubic angle (*i*.*e*. the angle formed by the inner edges of the ischiopubic branches below the pubic symphysis), and the length of the ischiopubic branch (*i*.*e*. the distance between the superior face of the pubis and the most distal part of the ischial tuberosity). We used the middle of the ischiopubic branch (MISP) and the middle of the outer edge of the obturator foramen (MOE)–*i*.*e*. the middle of the distance between horizontal branch of the pubis and ischiopububic ramus within the obturator foramen–as fixed landmarks in each patient to delineate the area. The ischiopubic branch is the bone branch of the bony pelvis that originates from the pubis and ends at the ischium. We then measured the distances between all the arteries of the obturator foramen and our landmarks: MISP to the bifurcation of the obturator artery (BOA); MISP to the anterior obturator artery (AOA); MISP to the posterior obturator artery (POA): and MOE to the posterior obturator artery (POA). All measurements were done by two investigators whenever the vessels were present. They were repeated three times, averaged, recorded and pooled for each side.

### Anatomic mapping and definition of an avascular area from angiotomodensitometry reconstructions

In order to delimit an area with ≤ 5% risk of encountering any vessel, we used the 5^th^ or the 95^th^ percentile of each measurement of the position of the vessel with respect to the bone from both the anatomic and angiographic data.

### Anatomic study

Bilateral dissection from 10 fresh female cadavers provided 20 obturator foramen for anatomic analysis. All subjects were Caucasians. The bodies had been donated to the Paris School of Surgery and the Department of Anatomy, University Paris Descartes for research. Details about the exact age, parity or cause of death were not available. However, we checked that no previous pelvic surgery, such as hysterectomy or prolapse repair, had been performed and that there were no obvious abdominal scars, anatomic abnormalities or evidence of disease in the pelvis. All available anatomic samples meeting these conditions were included.

The cadavers were prepared as described in our previous work[16]. Five cadavers were injected with intravascular blue dye to better identify and follow arteries. The dissections were performed in the dorsal decubitus position for the abdominal approach and the gynecologic position for the perineal approach. We registered anatomic descriptions and observations.

## Results

### Radiologic findings

The shape of the obturator foramen was triangular most of the time and we found various forms and sizes of bone delimiting the structure. The mean (SD) interforaminal distance was 57.1 (4.0) mm. The mean (SD) pubic angle was 98.5 (9.4) degrees. The mean (SD) area of the obturator foramen was 11.0 (1.3) and 10.8 (1.8) mm on the right and left side, respectively. The mean (SD) length of the ischiopubic branch was 72.4 (7.2) and 72.3 (6.1) mm on the right and left side, respectively. The MISP was so located at 36.2 and 36.1 mm on the right and left side respectively. The mean (SD) length of the outer edge of the obturator foramen was 44.6 (4.6) and 45.0 (4.3) mm on the right and left side respectively. The MOE was so located at 22.3 and 22.5 mm on the right and on the left respectively.

**Fig 2:**
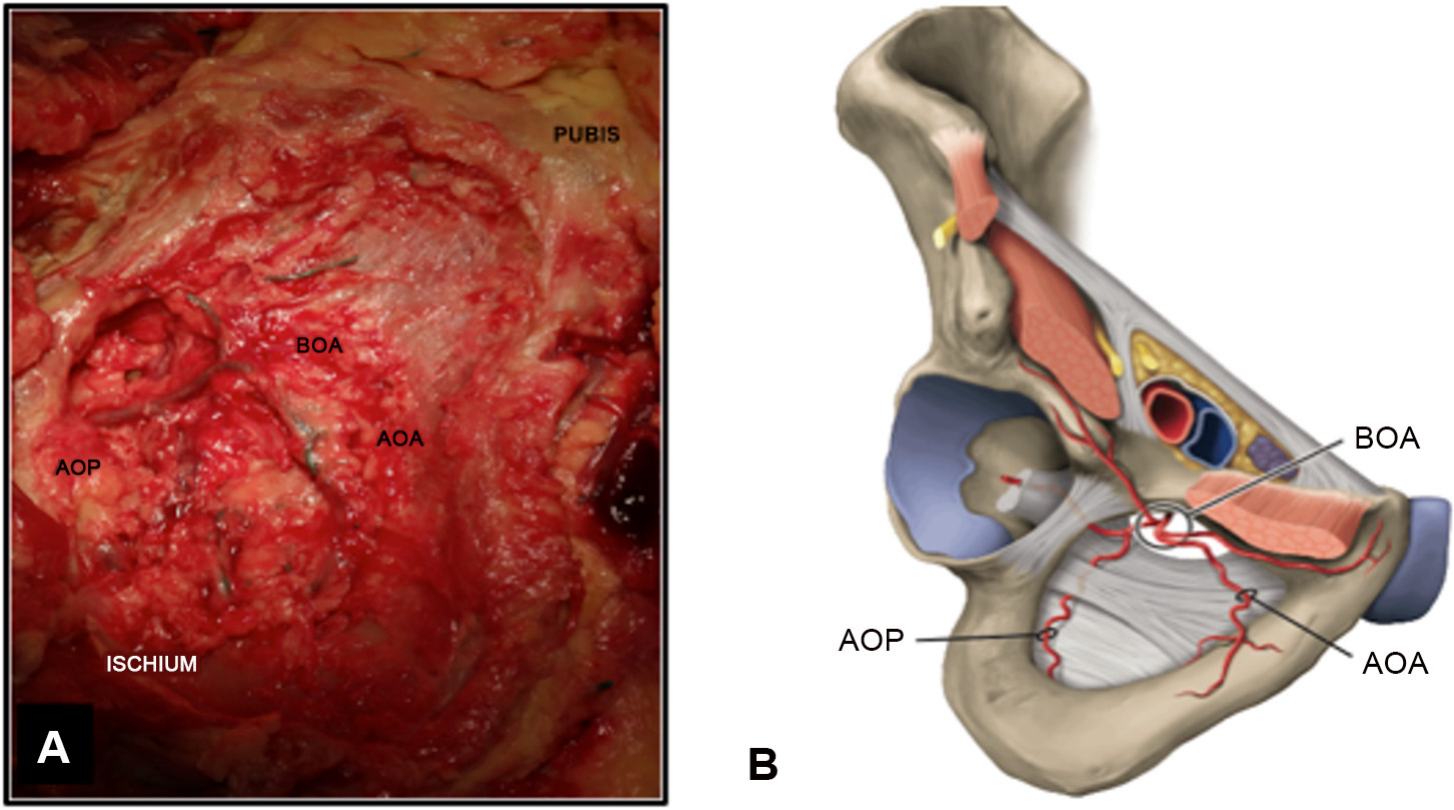
A. Obturator arteries reconstructions by angiotomodensitometry. The posterior obturator artery (POA), anterior obturator artery (AOA) and obturator artery bifurcation (BOA) are reconstructed. B. Triangular avascular area within the obturator foramen. A triangle avascular area exists between anterior and posterior obturator artery. Point A represents obturator artery bifurcation. Point B is at mean distance between MISP and anterior obturator artery. Point C is at mean distance between MOE and posterior obturator artery. MISP: Middle of the IschioPubic branch. MOE: Middle of the Outer Edge of the obturator foramen.

We observed complete anterior or posterior obturator arteries in 88.46% (n = 92) of cases (Fig 2A). From a purely visual assessment, the caliber of the vessels varied. The arteries were thin or absent in 12.50% (n = 13) and 8.65% (n = 9) for the anterior and the posterior branch, respectively. The mean (SD) distance between MISP–AOA was 15.1 (10.8) mm on the right side and 15.3 (10.0) mm on the left; the mean (SD) distance between MISP–BOA was 29.1 mm (3.6) for the right side and 30.2 mm (5.2) for the left; the mean (SD) distance between MOE–POA was 5.2 (3.3) mm on the right side and 5.7 (4.6) mm on the left; the mean (SD) distance between MISP–POA was 22.6 (4.4) mm on the right side and 24.6 (11.3) mm on the left (Table 1). An avascular area emerged from these measurements and was comprised between the 5^th^ and 95^th^ percentile of these measurements. The mean or median 5^th^ and the 95^th^ percentile of the distance between MISP–AOA was 0 and 32.1 mm on the right side respectively and 1.8 and 26.8 mm on the left respectively; for the distance between MISP–BOA it was 24.2 and 35.1 for the right side respectively and 24.2 and 36.4 mm for the left, respectively. The mean or median 5^th^ and the 95^th^ percentile of the distance between MOE–POA was 0 and 12.3 mm on the right side respectively and 0 and 13.9 mm on the left, respectively. The mean or median 5^th^ and the 95^th^ percentile of the distance between MISP–POA was 15.8 and 30.0 mm on the right side respectively and 16.3 and 29.8 mm on the left, respectively. This avascular area had a triangular shape and was in the center of the obturator foramen between the posterior and anterior obturator artery (Fig 2).

**Table 1:** Distance between foraminal obturator arteries using fixed bony landmarks from angiotomodensitometry reconstructions.

	MISP-AOA^a^		MISP-BOA^b^		MOE-POA^c^		MISP-POA^d^	
Patient	Right	Left	Right	Left	Right	Left	Right	Left
**Mean**	15.1	15.3	29.9	30.2	5.2	5.7	22.6	24.6
**Min**	0	0	21.6	23.8	0	0	14.2	4.1
**Max**	33.6	39.4	40.3	59.7	15.5	21	30.5	94.8
**Standard deviation**	10.8	10.0	3.6	5.2	3.2	4.6	4.4	11.3
**5th percentile**	0	1.8	24.2	24.2	0	0	15.8	16.3
**95th percentile**	32.1	26.8	35.1	36.4	12.3	13.9	30.0	29.8

^a^MISP-AOA: distance between the middle of the ischiopubic branch (MISP) and the anterior obturator artery (AOA)

^b^MISP- BOA: distance between the middle of the ischiopubic branch (MISP) and the bifurcation of the obturator artery (BOA)

^c^MOE-POA: distance between the middle of the outer edge of the obturator foramen (MOE) and the posterior obturator artery (POA)

^d^MISP-POA: distance between the middle of the ischiopubic branch (MISP) and the posterior obturator artery (POA).

### Anatomic findings

Twenty obturator regions were available for anatomic study. After removal of the skin, subcutaneous layer of the thigh and the fascia lata, we identified three muscular layers inserted in the obturator foramen bone. The superficial layer was composed of the gracilis muscle. The middle layer was composed of the adductor longus, adductor brevis and the adductor magnus muscles. Finally, the deep layer was composed of the externus obturator muscle. Removal of the muscles including the external obturator muscle revealed the obturator area and its neurovascular structures.

**Fig 3:**
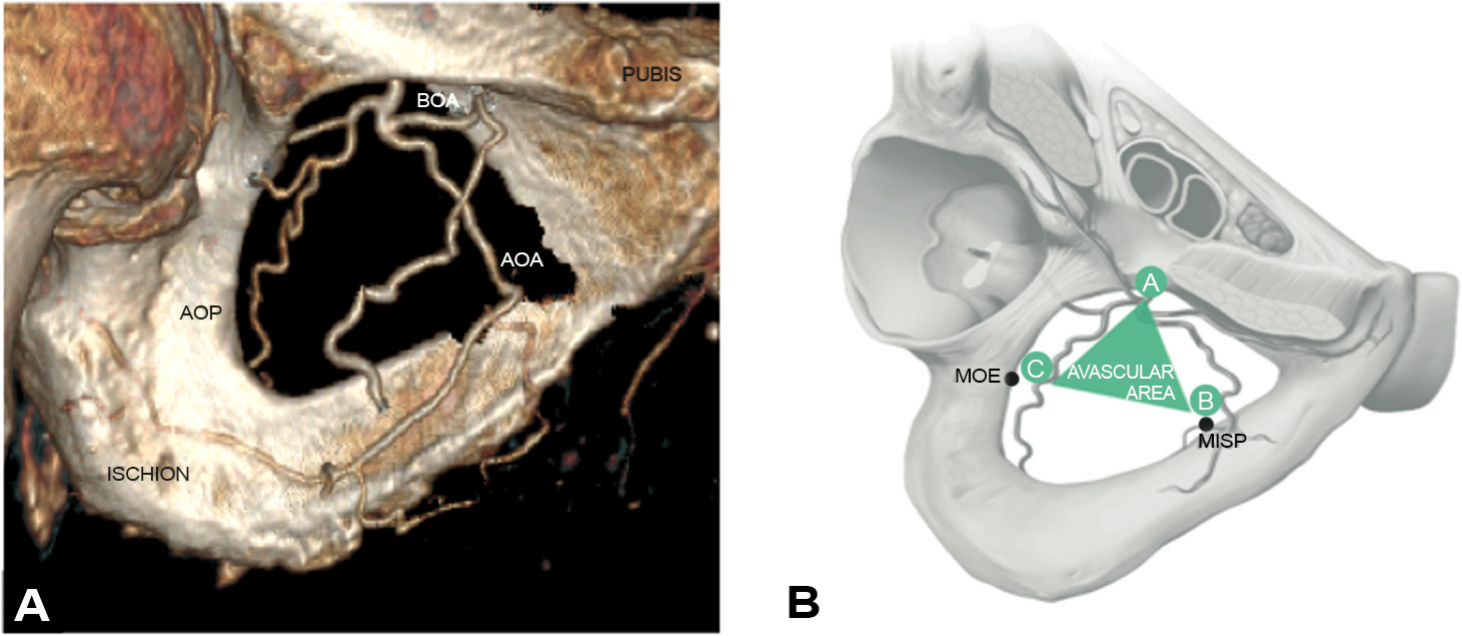
A. Anatomic perineal view of obturator foramen. B. Schematic perineal view of obturator foramen. The posterior obturator artery (POA), anterior obturator artery (AOA) and obturator artery bifurcation (BOA) are represented. POA and AOA form a circle.

The obturator vessels ran under the obturator nerve arising from the anterior division of the internal iliac vessels by abdominal approach. The obturator pedicle (nerve, artery and vein) crossed the obturator canal located at the cranial and ventral edge of the obturator foramen. The anterior and posterior branches of the obturator vessels were identified on the obturator membrane, running along the bone and describing a circle by perineal approach (Fig 3A). The central triangular part of the obturator area was free of vessels (Fig 3A and 3B).

Anterior and posterior obturator nerves were localized above obturator artery. They had a supero-external pathway and so were not localized on the avascular triangle.

## Discussion

Our data using fixed landmarks in angiotomodensitometry reconstructions suggest that beyond inter-individual variations of the obturator foramen, a central triangular avascular area exists within the obturator foramen between the anterior and posterior obturator artery (Fig 2B). The bifurcation of the obturator artery was at a mean distance (SD) of 29.2 (3.6) and 30.2 (5.2) mm of the MISP on the right and left side respectively. The anterior branch of the obturator vessels was at a mean distance (SD) of 15.1 (10.8) mm and 15.3 (10.0) mm of the MISP on the right and left side respectively. The posterior branch of the obturator vessels was at a mean distance (SD) of 5.2 (3.2) mm and 5.7 (4.6) mm of the MOE on the right and left side respectively. Finally, the posterior branch of the obturator vessels was at a mean distance of 22.6 (4.4) mm and 24.6 (11.3) mm of the MISP on the right and left side respectively (Table 1).

To our knowledge, there are few data about the obturator arteries within the obturator foramen [18–22] (MEDLINE 1966-Octobre 2015: English language; search terms: obturator foramen, obturator artery, angiotomodensitometry). Our study is original in that it defines a safe avascular area for transvaginal mesh placement and provides objective mapping and measurements of the obturator vessels. Furthermore, we describe the obturator anatomy with fixed bone landmarks. The strength of our study is that it is based on both anatomic and radiologic data allowing correlation to correctly interpret pre-operative imaging. The avascular area was described both by anatomic dissection and CT scan. Anatomic descriptions of the obturator region give only descriptive information but helped us to interpret the angiotomodensitometry data. These reconstructions add objective measures and allowed an objective mapping of the obturator regions. Due to the high number of CT scan values, we were able to apply a volumetric-rendering technique. This resulted in a reliable reconstruction of the vascular environment—as precise as 250 μm sections for an accurate resolution–and is thus highly relevant for routine practice. Radiologic analysis also has the advantage of being operator-independent and avoiding issues such as dissection artifacts, which could modify the vascular anatomy and relation to the neighboring structures.

However, there are limitations to our study. The population included in the radiologic study was more likely to have arteritis than the general population because it would not have been ethically justified to perform angiotomodensitometry without a medical reason. In fact, angioscannography was indicated for a suspicion of arteriopathy in most of the cases. This could infer that the vessel caliber in our series was thinner than in the general population which could modify the distance between the artery and the anatomical landmarks. It may also explain the rate of thin or absent arteries in the series. However, for 92 patients (88.46%) in whom arteries could be identified, the location was not modified and this should not therefore impair data interpretation. Furthermore, the avascular area seems to be bigger than a triangle and we could have defined an ellipse mainly for the lower part of the obturator area. However, this would have required useless and fastidious work with many measurements whereas the triangle is adequate and focused on the upper part, which is at vascular risk. Another limitation could lie in the small number of dissected cadavers. Nevertheless, all dissections were informative. Obturator vessels were always identified and our findings confirm previous classical anatomic studies [16,18–22]. Moreover, the dissections were made under operative conditions, improving the value of the data.

Some major anatomic studies of the obturator foramen have already been performed to describe the relation between neurovascular structures and transobturator devices [16,18–22]. The two main studies reported the distance between the obturator vessels and the devices used during vaginal mesh placement for prolapse repair [16,22]. They found that the needle passed very close to the vessel and even touched them in some cases [16,22]. This emphasizes the need for precise vascular mapping of the region. The needles are placed after dissection of the vagina until they reach the medial part of the ischiopubic branch, which is the fixed surgical landmark. We show that there is an avascular area between the two branches of the obturator vessels and its position with respect to the middle of the ischiopubic branch, one of the only fixed landmarks palpated by the surgeon. Although some radiologic studies have investigated this region, none have localized the foraminal obturator artery branches. Bogusiewcz et al. [23] performed pelvic computer tomography on 122 women and measured the distance between the interobturator foramina line and inferior pubic symphysis. They report a high variability of the pelvic bone architecture and conclude that it may play a role in transobturator tape positioning.

To date, surgeons using the transobturator approach usually perform surgery based on palpation only and only have the ischiopubic bone branch and ischial spine as landmarks that can be palpated during the procedure. However, knowledge of the location of neurovascular structure is crucial to reduce the risk of complications. This study may therefore help them gain better knowledge of vascular pitfalls and orient them toward secure areas within the obturator foramen.

## Conclusion

We have defined a central triangular avascular area within the obturator foramen, located between the two branches of the obturator vessels. This avascular area is based on the distances of the vessels with fixed bone landmarks of the obturator foramen. Our findings could be of value to reduce vascular complications during transobturator vaginal mesh techniques for prolapse repair. Future anatomic studies could further describe the vascular obturator region to avoid palpation pitfalls.

## Supporting Information

S1 Table Obturator area measurements. (XLS)
